# An Efficient Method for Generating Poxvirus Recombinants in the Absence of Selection

**DOI:** 10.3390/v3030217

**Published:** 2011-03-09

**Authors:** Amanda D. Rice, Stacey A. Gray, Yu Li, Inger Damon, Richard W. Moyer

**Affiliations:** 1 Department of Molecular Genetics and Microbiology, University of Florida, 1600 SW Archer Rd., Gainesville, FL 32610, USA; E-Mails: gray.staceya@gmail.com (S.G.); rmoyer@ufl.edu (R.M.); 2 Poxvirus and Rabies Branch, Centers for Disease Control, 1600 Clifton Rd., Atlanta, GA 30333, USA; E-Mails: lay4@cdc.gov (Y.L.); iad7@cdc.gov (I.D.)

**Keywords:** poxvirus, recombinant, vaccinia virus, helper virus, fowlpox virus

## Abstract

The use of selectable markers (*ecogpt*) and selection pressures to aid in detection of poxvirus (Vaccinia, VV) recombinants has been implicated in the unintended introduction of second site mutations. We have reinvestigated the use of the helper virus system described by Scheiflinger *et al.* [1] and adapted by Yao and Evans [2] which produces recombinants at a high frequency in the absence of any selection, at a rate of 6–100%. Our system uses fowlpox virus (FPV) as the infectious helper virus which in infected cells provides the enzymatic apparatus for transcription and replication of a purified, transfected VV genome and for recombination with a second transfected PCR generated DNA fragment. To optimize the system, a PCR DNA fragment was generated that contained poxvirus promoter driven *gfp* and *lacZ* genes inserted within the coding sequences of the viral thymidine kinase gene. This PCR fragment was co-transfected together with VV genomic DNA. Recombinant VV was identified by plaquing the mixture on cells non-permissive for FPV and selection of green fluorescent or LacZ positive recombinant vaccinia plaques. The system was optimized using FPV permissive cells (CEF) and non-permissive cells (A549, CV-1) for both the initial infection/transfection and the subsequent selection. Up to 70% of the progeny vaccinia virus contained the *gfp/LacZ* insertion. In order to test for the presence of FPV/VV intertypic recombinants or other unintended mutations, recombinant wtVV (RwtVV) was regenerated from the *gfp/LacZ* viruses and evaluated by RFLP analysis and pathogenesis in animals. While all RwtVVs were viable in cell culture, in many of the RwtVV isolates, RFLP differences were noted and while some recombinant viruses exhibited wild type behavior in mice, a wide range of virulence indicative of unintended changes suggests that mutants created by “rescue” systems require careful analysis particularly before use for *in vivo* studies employing animal models.

## 1. Introduction

The generation of recombinant poxviruses is essential for determining the role of individual genes in virus growth and pathogenesis. This technology is critical for the field of poxvirus biology and thus a number of elegant reports have addressed the most efficient method of generating recombinant viruses [2–6]. Although there is a long history of fine tuning the methodology for generating recombinant poxviruses, the same protocols with few modifications have been used for decades [2,3,5,7–10]. The fine tuning has involved the optimization of length of homologous nucleotide sequences necessary for recombination to occur and the benefits of linear DNA as compared to plasmid DNA used in the reactions [9–17].

Historically, the generation of recombinant poxviruses has been labor and time intensive. The historical method for generating recombinant orthopoxviruses involved transfecting plasmid DNA containing the target mutation in cells infected with the parent virus, allowing for homologous recombination to occur and then purifying the desired virus. This method required not only the generation of the plasmid containing the target mutation but multiple rounds of plaque purification screenings to produce the desired virus recombinant. Such methods for generating recombination in a nonessential gene can be relatively inefficient, generating below 1% recombinant viruses at the beginning of the plaque purification screening [11,15]. This low rate of recombination has been observed out of the infection/transfection reaction regardless of the manipulation of variables such as the viral multiplicity of infection (MOI) and amount of plasmid DNA transfected.

The low rate of recombinant virus generation can be increased dramatically by the use of selectable markers such as *ecogpt* [18–22]. Selectable markers are advantageous in the ease and time required in which recombinant viruses can be identified. However, the use of selectable markers has been implicated in causing secondary mutations elsewhere in the genome not obvious until the virus revertant is generated and analyzed [23]. While viruses with secondary site mutations may not exhibit altered properties in a cell culture based system, those mutations may produce unexpected phenotypes when such viruses are used in animal models.

Previous helper virus systems have used orthopox, leporipox and avipox viruses as helper viruses with transfected fragmented target rescue virus genomes and a linear DNA fragment containing the desired mutation [1,2,24,25]. These systems relied on the helper virus to provide the machinery necessary to assemble the fragmented genome while simultaneously integrating the targeted mutation. While the rate of recombinants generated was near 100% in these studies, the generation of digested fragmented virus genomes is labor intensive and requires the desired mutation to fall near a restriction enzyme site for efficient recombinant virus generation [1,2,24,25]. Therefore, each recombinant virus requires a great deal of *in silico* strategy and planning before attempting to generate the desired virus. In an era that values the high throughput systems this procedure is not desirable and outdated.

Here we describe a system similar to that used by Yao and Evans [25] to generate high rates of recombinant viruses. This system uses fowlpox virus (FPV) as the helper virus in infected cells which are transfected with intact vaccinia virus (VV) wildtype genomic DNA together with a PCR generated DNA fragment containing the mutation of choice. The FPV helper virus system supplies the enzymatic machinery needed for recombination and also allows rescue and production of infectious VV [1,26,27]. FPV was chosen to rescue orthopoxvirus genomes due to its narrow host range [28], infecting mammalian cells but only replicating in avian cells, allowing for elimination of FPV from the orthopoxvirus mixtures by plaquing on mammalian cells.

To avoid the standard time consuming procedures required to generate recombinant orthopoxviruses via the classical method and the problems implicated by the use of selectable markers in generating recombinant viruses, we sought a simple, reproducible method for generating recombinants at a high frequency without the use of selection pressures. The procedure we describe results in generation of desired mutations at a frequency of 70%. While generating high levels of recombinant viruses quickly is advantageous, we further took steps to analyze whether the resulting virus isolates exhibited unintended mutations and pathogenesis which differed from that of the parent VV.

## 2. Results and Discussion

### 2.1. Generation of VV in FPV Permissive cells

**Figure 1.**
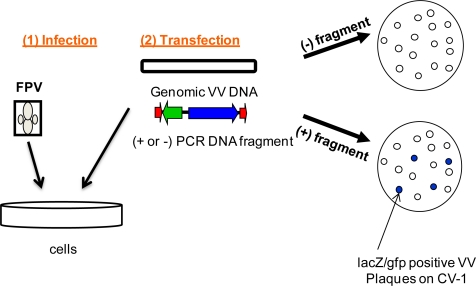
Infection/transfection scheme for generation of recombinant vaccinia virus. Cells are infected with fowlpox virus (FPV) and transfected with genomic vaccinia virus (VV) DNA ± a disrupted VV PCR fragment containing the desired mutation (*lacZ* or *gfp*). The resulting virus mixture is then plaqued on mammalian cells to eliminate FPV and the desired viruses isolated.

To evaluate and optimize the helper virus system, we began by assessing the ability of fowlpox virus (FPV) to produce infectious vaccinia virus (VV) from FPV infected cells transfected with VV genomic DNA with or without transfected VV PCR generated DNA fragments. The infection/transfection rescue scheme for all conditions tested in this report is depicted in Figure 1. Our initial transfections were done in chicken embryo fibroblast (CEF) cells which are permissive for FPV growth [28].

**Figure 2.**
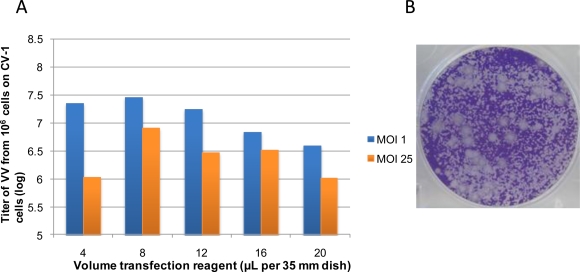
Optimization of transfection reagent for rescue of infectious vaccinia virus particles. **(a)** Titers of VV rescued from chicken embryo fibroblast (CEF) cells infected with FPV at two different FPV MOI and transfected with 12 μg VV genomic DNA using varying amounts of transfection reagent Lipofectamine 2000. **(b)** Crystal Violet staining of the initial infection/transfection mixture plaqued on CV-1 cells. Plaquing on CV-1 cells of VV rescued from CEF cells infected with FPV at an MOI of 25.

In order to optimize the generation of recombinant VV using FPV, we first ensured that we could obtain viable VV from this helper virus system, and optimized the yield of VV prior to generating any recombinant viruses. The first condition optimized was the ratio of transfection reagent to DNA in FPV infected cells. CEF cells were infected with FPV at an MOI of 1 or 25 and transfected with 12 μg of intact genomic VV DNA while varying the amount of cationic transfection reagent Lipofectamine 2000. Lipofectamine 2000 serves to facilitate the DNA delivery and uptake via endocytosis [29,30]. A 1:1 ratio of total μg DNA to μL of transfection reagent was ultimately confirmed as the best DNA:detergent ratio for all future experiments, and this ratio was in agreement with manufacturer recommendations (Figure 2a).

There were few differences in the total virus amount recovered between the reactions using 4, 8 or 12 μL of Lipofectamine 2000 at either FPV multiplicity of infection. At high volumes of transfection reagent, the virus titer decreased slightly, most likely caused by cytotoxicity typical of high doses of cationic lipids [31–33]. In our hands, 12 μL of the transfection reagent together with 12 μg of total DNA was selected as optimal for all future experiments.

When the cells were infected with FPV at an MOI of 1, the total VV recovered was somewhat higher than when the cells were infected at an MOI of 25 (Figure 2a). However, this increase may be attributed to a secondary amplification of rescued VV leading to infection of cells not initially infected by FPV at the lower multiplicity. In order to ensure that all the cells in the cell monolayer were initially infected by FPV while minimizing any secondary amplification of VV, FPV at an MOI of 25 was used for all future experiments.

When an infection/transfection mixture harvested from CEF cells was plaqued on a cell line nonpermissive to FPV such as CV-1 cells and stained with Crystal Violet, a total of over 10^7^ plaques were observed consisting of both large and small plaques (Figure 2b). Typically, the ratio of large to small plaques was roughly 2:1 upon plaquing of the initial infection/transfection mixture on CV-1 cells. The large plaques were typical of wtVV plaques. However, the heterogeneity could have arisen in part from FPV/VV intertypic recombinants, partially defective VV or plaques of mixed genotypes. Visualization of the infection/transfection mixture via plaque assay demonstrated that a majority of plaques, independent of size, were GFP positive. Upon sequential passage on a FPV nonpermissive cell line, CV-1, the number of small plaques decreased and eventually disappeared all together by the second or third passage. Plaques most similar to those of wtVV were chosen for further studies.

### 2.2. Generation of Recombinant VV

The system was next evaluated for the ability to generate high levels of VV recombinants as a function of genome/PCR DNA fragment ratios. To determine rates of recombination, a PCR-generated 3,438 bp fragment containing *gfp* and *lacZ* under the poxvirus P_E/L_ and P_7.5_, respectively, flanked by TK gene sequence was used. The *gfp* and *lacZ* genes allow for ready detection of recombinants [3,6,7,34].

The process of optimizing the generation of recombinant VV was performed using conditions optimal for FPV mediated rescue of VV as described and shown in Figure 2. CEF cells were infected with FPV at an MOI of 25 and co-transfected with a total of 12 μg DNA, using genomic VV DNA and *gfp/lacZ* PCR fragment at varying ratios. Mass ratios of genomic DNA to fragment DNA of 1:20, 1:10, 1:1, 10:1 and 20:1 were used to determine the optimal ratio for generation of recombinant VV. The resulting infection/transfection mixtures were plaqued on CV-1 cells and analyzed for the number of LacZ positive plaques relative to the total number of plaques obtained.

**Figure 3.**
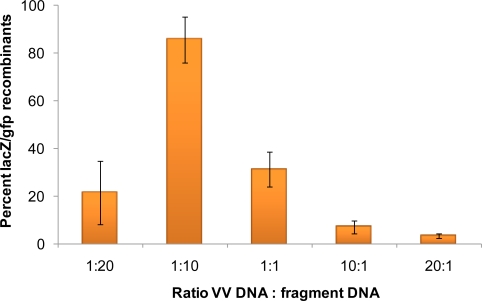
Frequency of VV *lacZ/gfp* recombinant viruses from optimization of ratio of genomic DNA to *gfp/lacZ* PCR fragment in CEF cells. Cells were infected with FPV at an MOI of 25 and transfected with a total of 12 μg DNA in the ratios noted in the graph. Error bars represent SEM; n = 10.

At all ratios tested recombinant VV was obtained (Figure 3), however the percent of recombinant VV was highly dependent on the ratio of VV DNA: *gfp/lacZ* PCR fragment. The highest numbers of recombinant VV were observed when the ratio of genomic DNA:*gfp/lacZ* PCR fragment was 1:10. At this ratio greater than 80% of the total resulting VV viruses were recombinants. While these ratios are based upon total mass of DNA added, the ratio of copy numbers is much higher, with the *gfp/lacZ* PCR fragment being in substantial excess compared to that of genomic VV copy number, and is theorized to force the recombination between the VV DNA and *gfp/lacZ* PCR fragment.

### 2.3. The Use of Cells Non-Permissive for FPV to Rescue VV

Although high numbers of infectious VV and/or VV recombinants were generated in FPV permissive cells, as was noted in Figure 2, the infection/transfection mixture produces heterogenous plaque sizes consistent with the formation of significant numbers of VV variants or even FPV/VV intertypic recombinants (Figure 1b). Therefore, a cell line that supported the generation of recombinant VV while limiting FPV growth was desirable.

**Figure 4.**
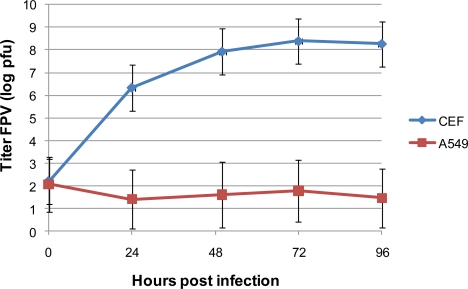
Growth of FPV on permissive CEF cells and non-permissive A549 cells. Cells were infected with FPV at an MOI of 0.001, harvested at 0, 24, 48, 72 and 96 hours post infection, and titered on CEF cells.

FPV has a limited host range restricted to avian cells but will infect mammalian cells in an abortive manner [8,35,36]. Based upon this host restriction, the mammalian cell line A549 was tested for support of FPV growth using a low multiplicity growth curve (Figure 4). There was no virus growth of FPV on A549 cells whereas the yield of FPV from CEF cells was 10^8^ pfu within 48 hours. Given that FPV does not replicate on A549 cells, we then tested whether A549 cells could be utilized for rescuing transfected genomic VV DNA and generating recombinant VV in an attempt to limit the amount of FPV produced during the infection/transfection.

**Figure 5.**
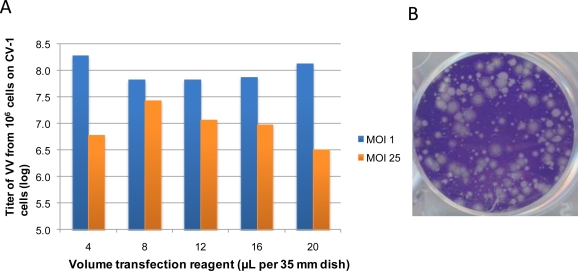
Optimization of transfection reagent for rescue of infectious vaccinia virus particles using A549 cells. A549 cells were infected with FPV at an MOI of 1 or 25 and transfected with genomic VV DNA at varying concentrations of Lipofectamine 2000. **(a)** Rescue of VV in A549 cells using varying amounts of transfection reagent and 12 μg VV DNA at a low (1) and a high (25) MOI of FPV. Error bars represent SEM. **(b)** Plaquing stained with crystal violet on CV-1 cells of resulting virus mixture from VV rescued in A549 cells infected with FPV at an MOI of 25.

Again, after confirmation that FPV infected A549 cells would support rescue of transfected genomic VV, the conditions for generating recombinant VV were optimized as initially described in Figure 1. Once again, we observed that FPV was readily able to rescue intact genomic VV DNA to produce infectious VV as indicated by the ability to plaque on CV-1 cells. The conditions were again optimized using A549 cells for the MOI of FPV and ratio of transfection reagent to total DNA transfected of genomic DNA (Figure 5a). The optimal conditions were the same as those determined for the CEF cells in which FPV at an MOI of 25 and a ratio of 1:1 v/mass for Lipofectamine 2000: genomic DNA.

**Figure 6.**
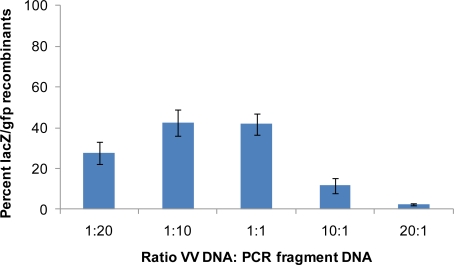
Frequency of VV *lacZ/gfp* recombinant viruses generated from FPV infected A549 cells. A549 cells were infected with an MOI of 25 and transfected with 12 μg of total DNA in the ratios shown using 12 μL of transfection reagent. The percentages of recombinant viruses were determined by staining for the presence of LacZ and counting the number of LacZ positive plaques in relation to the total number of plaques. Error bars represent SEM. N = 4.

We then analyzed the ability of FPV infected A549 cells to produce VV recombinants following transfection with both VV intact DNA and the PCR fragment encoding *gfp/lacZ* in the same fashion as shown in Figure 2. These results are shown in Figure 6. As in CEFs, the maximum percentage of recombinant viruses was obtained when using a mass ratio of 1:10 genomic VV DNA to *gpf/lacZ* PCR fragment DNA, with an average yield of 50% rescued viruses being recombinant VV. Importantly, infection/transfection of A549 cells produced far lower backgrounds of small plaques than were observed in infected/transfected CEF cells (compare Figures 2b and 5b) with 2 × 10^5^ VV plaques and 4 × 10^5^ small plaques observed upon plaquing of the infection/transfection reaction. By using A549 cells for the infection/transfection, the number of small plaques was decreased by 10-fold allowing for better visualization and separation of the desired plaques. The comparative differences in plaque morphology between CEF cells and A549 cells indicate a clear cell line dependence on outcome.

### 2.4. Analysis of Resulting Viruses for Unintended Mutations and Pathogenicity

Although many of the presumed vaccinia viruses generated using the FPV helper virus system produced plaques similar in size to those of wtVV and behaved as such in cell culture, more stringent tests were needed to ascertain whether these recombinants were truly “wild type virus” in all respects except for the newly introduced gene. To address this issue, the recombinant viruses containing the *gfp/lacZ* marker gene were reverted back to wild type using the FPV system. This was accomplished by taking several individual VV recombinant isolates derived from different infection/transfection reactions and first purifying the recombinant VV DNA using the same procedures as for the wtVV DNA. This recombinant VV DNA was then used in the FPV mediated infection/transfection system of infected A549 cells as described above together with a PCR generated wild type TK gene fragment. The resulting viruses are referred to as rescued recombinant wtVV (RwtVV) and were plaque purified, selecting for the absence of *gfp/lacZ* and used for pathogenesis as well as an analysis of differential plaquing efficiency and random alterations in overall sequence by restriction length polymorphism (RFLP) analysis [37]. It is important to note that all viruses were easily isolated, requiring only three rounds of plaque purification.

Nine separate RwtVV isolates were used for *in vivo* analysis of virulence in C57BL/6 mice. Groups of three animals were inoculated via intratracheal injection with 10^6^ and 10^7^ pfu for each of the RwtVV isolates and compared to animals infected with wtVV. Animal health was assessed daily including body temperature, weight loss and overall appearance. Animals were euthanized if their body temperature decreased from a normal of 36 °C to 30 °C or lower or if they experienced weight loss of greater than 30% of initial body weight. Typically, a dose of 10^6^ pfu of wtVV is 100% lethal in C57BL/6 mice, accompanied by significant weight loss and other clinical symptoms necessitating euthanasia within 6 to 8 days post infection [38].

The RwtVV *in vivo* phenotypes relative to wtVV phenotypes are reported in Table 1. Two of the nine isolates (RwtVV 3 and 6) exhibited a phenotype identical to that of wtVV with all animals at both 10^6^ and 10^7^ pfu doses requiring euthanasia due to severe disease. Three of the nine isolates exhibited attenuation at the 10^6^ pfu dose but lethal disease at the 10^7^ pfu dose, thereby being classified as viruses that were attenuated by 1 log (RwtVV 5, 8 and 9). RwtVV 7 was classified as 2 logs attenuated in which all the mice at the 10^6^ pfu dose were relatively unaffected while the animals at 10^7^ pfu exhibited moderate disease. Three of the nine viruses were completely attenuated with all animals at both doses exhibiting few or no disease symptoms at all (RwtVV 1, 2 and 4).

Given the variation of the RwtVV virus isolates in mice, further *in vitro* examination of the isolates was performed. The goal was to determine if the relative pathogenesis in animals could be predicted via cell culture based assays as a time and cost effective evaluation of the produced viruses. For this assay, we evaluated relative plaquing efficiencies of the viruses on mildly restrictive CV-1 cells and highly restrictive PK-15 cells, which are among the most restrictive cell lines for vaccinia virus, tolerating fewer deleterious mutations than other cell lines routinely utilized [39]. All the RwtVV isolates that exhibited a phenotype comparable to that of wtVV had CV-1:PK-15 plaquing ratios of less than 50, similar to that of wtVV (Table 1). The ratios of all the other viruses did not correlate well with pathogenesis predictions in mice; several completely apathogenic isolates (Rwt1 and 4) had relatively low ratios of 80 and 275, respectively, while RwtVV 8 exhibited 1 log attenuation in mice and had a ratio of 9074. These results suggest that recombinant viruses that exhibit relative plaquing ratios greater than that of wtVV are at a higher risk for behaving aberrantly in an *in vivo* evaluation.

**Figure 7.**
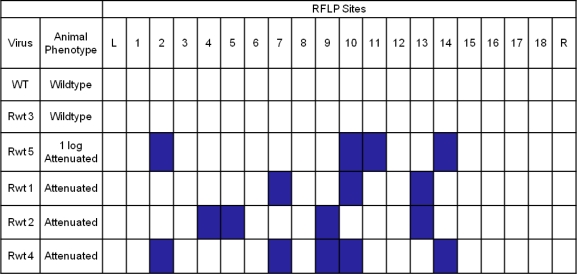
Restriction fragment length polymorphism (RFLP) analysis of rescued wild type VV. The genomes of the viruses noted in the table underwent RFLP analysis as compared to the parental wild type virus. Any changes in banding patterns were noted as a change in that section of the genome. Changes for the five selected isolates are noted by a dark blue shadowing of the box, no shading represents no changes were detected. For comparison purposes the phenotype of the viruses in animals is also shown on the table as previously shown in Table 1.

RwtVV 1 to 5 were also examined by RFLP analysis at the Center for Disease Control to detect changes in genome sequence that could account for the variation in pathogenicity observed. The RFLP analysis examined the genomes via restriction digestion patterns of 20 segments crossing the genomes. All RwtVV isolates that exhibited attenuation in mice had significant polymorphisms compared to wtVV, with locations exhibiting any alteration in RFLP pattern notated as a region with changes (Figure 7). While there were some locations with less significant changes, again there was no clear correlation of banding patterns to pathogenesis. RwtVV 1, 4 and 5 exhibited changes at fragment 10, which is the band in which the TK gene is located, indicating the change occurred during recombination. The *gfp/lacZ* viruses from which the RwtVV isolates were derived also underwent RFLP analysis and there were few changes observed except for the expected change in fragment 10 where the TK gene is located, suggesting that the changes accumulate over time with contributions from both normal passage of virus in cell culture and recombination with FPV (data not shown). While the changes observed in the RFLP analysis were presumed to be due in part to recombination with FPV it is not possible to determine which changes were due to routine passage in cell culture.

## 3. Experimental Section

### 3.1. Cell Culture

CV-1 and PK-15 cells were maintained in Minimum Essential Media (MEM) with Earle’s Salts (Gibco, Grand Island, NY, USA) supplemented with 2 mM glutamine (Media Tech, Herndon, VA, USA), 50 U/mL penicillin G and 50 μg/mL streptomycin (Media Tech), 1 mM sodium pyruvate (Media Tech), and 0.1 mM nonessential amino acids (Media Tech) and 5% v/v FBS (Gibco).

CEF cells were maintained in Media 199 supplemented with 2 mM glutamine (Media Tech, Herndon, VA, USA), 50 U/mL penicillin G and 50 μg/mL streptomycin (Media Tech), 1 mM sodium pyruvate (Media Tech), and 0.1 mM nonessential amino acids (Media Tech), and 10% v/v FBS (Gibco).

A549 cells were maintained in Dulbeco’s Modified Eagle Medium supplemented with 2 mM glutamine (Media Tech, Herndon, VA, USA), 50 U/mL penicillin G and 50 μg/mL streptomycin (Media Tech), 1 mM sodium pyruvate (Media Tech), and 0.1 mM nonessential amino acids (Media Tech), and 10% v/v FBS (Gibco).

### 3.2. Virus Propagation

VV-WR (wild type VV), VV*gfp/lacZ* and RwtVV were grown and titered on CV-1 or PK-15 cells using standard methods [40,41]. FPV-T7 was grown and tittered on CEF cells using standard methods [42]. All viruses for animal infections were pad purified over 36% sucrose using standard methods and resuspended in PBS [40,41].

### 3.3. DNA Isolation from Virus

Virus was placed onto confluent CV-1 150 mm dishes at an MOI of 0.025. The virus was harvested 4 days post infection by centrifugation at 1430 x g at 4 °C for 10 minutes. The pellet was resuspended in 14 mL 14 mM Tris-Cl pH 7.5. The cells were mechanically homogenized via ∼ 40 strokes of the dounce and centrifuged at 300 x g for 5 minutes at 4 °C. The resulting supernatant was saved and 2 mL of 10 Mm Tris-Cl pH7.5 was used to resuspend the pellet. The resuspended pellet was centrifuged at 300 x g for 5 minutes at 4 °C and the resulting supernatant removed.

The supernatant underwent protein digestion with 2.45 × 10^−7^ mol 2-mercaptoethanol, 0.057 mg Proteinase K, 0.114 mmol sodium chloride, and 0.57% sodium dodecyl sulfate at 37 °C for 12 hours. The resulting mixture was then extracted twice with one volume of STE saturated phenol:chloroform:isoamyl alcohol. Three times the total volume of 200 proof ethanol and 0.3 mM sodium acetate pH 7.0 was added. The DNA was then swirled out using a heat sealed sterile pasteur pipette and suspended in sterile water.

### 3.4. Generation of PCR Fragments

A plasmid containing *gfp* under the P_E/L_ promoter and *lacZ* under the P_7.5_ promoter flanked by the vaccinia virus thymadine kinase gene (TK) named pSC65 gfp lacZ was used to generate the *gfp/lacZ* containing PCR fragment. This plasmid is a modification of pSC65 from the B. Moss laboratory [43]. PCR was carried out on pSC65 gfp lacZ using 1.6 U Vent DNA polymerase, 0.3 μM of each primer, 0.5 mM dNTP, and 0.2 μg plasmid DNA, and Vent ThermoPol Buffer (New England Biolabs, Massachusetts, USA). Thermocycler conditions were: 94 °C for 3 min; 10 cycles of 94 °C for 15 seconds, 53 °C for 30 seconds, 68 °C for 5 min; then 30 cycles with no changes except for the addition of 5 seconds per cycle to the 68 °C step; 68 °C for 7 minutes [44]. The primers used for generation of the PCR fragment from the plasmid were IDT131: GGCGGACATATTCAGTTG and IDT132: AGTCGATGTAACACTTTC. The overlapping TK gene sections were 250 bp PCR products were purified via Edge Biosystems PCR clean up columns per the manufacturer instructions prior to use in the transfection reactions.

The wild type TK gene fragment was amplified from VV-WR genomic DNA using IDT131 and IDT132 and subjected to the same purification steps as outlined for the *gfp/lacZ* PCR fragment.

### 3.5. Verification of gfp/LacZ in TK Locus

DNA of VV*gfp/lacZ* and RwtVV viruses was isolated using Qiagen DNeasy (Qiagen, California, USA) kit per manufacturer directions. PCR of the TK locus was performed using IDT131 and IDT132 for the presence of a wild type TK band (from wild type genes) of ∼ 500 bp and/or *gfp/lacZ* in the TK locus (from knockout viruses) of ∼ 5 kbp. The presence of the *gfp*/*lacZ* band with no wild type TK band by PCR and positive for LacZ and/or GFP was determined sufficient for confirmation of the desired virus construct. Alternatively, the presence of a wild type TK band only by PCR and the absence of GFP and LacZ by plaque analysis staining were determined to be sufficient for confirmation of a wild type TK locus virus (data not shown). Should the *gfp/lacZ* TK fragment had integrated into alternative sites in the genome generating revertant viruses would have been difficult to generate. This was not observed therefore no further analysis or sequencing was performed.

### 3.6. Infection/Transfection

FPV-T7 was inoculated onto CEF cells passage 2 or A549 passage 10 cells (2 × 10^6^ cells per 35 mm dish) at an MOI of 1 or 25 in an inoculum volume of 0.5 mL. After one hour of absorption the virus inoculum was removed and 0.1 mL transfection mixture was added followed by 1 mL of growth media without supplements. After 4 hours of incubation in incubators 1ml of complete media was added to each infection/transfection reaction. Twenty four hours post infection the mixture was harvested, centrifuged at 1300 x g for 20 minutes, and the cell pellets were resuspended in 0.3 mL DMEM with 1% HEPES.

### 3.7. FPV Growth Curve

Confluent A549 and CEF cells were inoculated with FPV-T7 at an MOI of 0.001 for 1 hour. The innoculum was removed and 2 mL of growth media added to the cells. Cells were isolated at times of 0, 24, 48, 72, and 96 hours post addition of growth media. The cell pellets were resuspended in media with HEPES and titered on CEF cells using standard methods.

### 3.8. Animal Infection and Monitoring

Infections were performed as previously described [38,45]. Briefly, mice were subjected to general anesthesia (isoflurane) prior to inoculation. All mice maintained a surgical plane of anesthesia during the procedure. A 3 mm ventromedial incision was made adjacent to the trachea for subcutaneous insertion of a microchip used to monitor body temperature and unique animal identification number (BioMedic Data Systems, Seaford, DE). A total volume of 30 μL of virus diluted in PBS was injected into the trachea. The incision was closed using surgical glue (Nexaband, Abbott Animal Health, Chicago IL).

Each animal was microchipped at the time of infection to transmit body temperature and identification number to the DAS-5007 reader (Bio Medic Data Systems). Weight, temperature, and physical observations of the mice (grooming habits, facial swelling, secretions, removal of hair, respiratory distress) were recorded daily. Criteria for euthanizing the mice include open mouth breathing, severity of dyspnea, hypothermia (less than or equal to 30 °C) or weight loss of greater than 30% of initial body weight. All animal procedures were carried out according to the University of Florida Institutional Animal Care and Use Committee guidelines.

### 3.9. RFLP Analysis of Vaccinia DNA

The details of vaccinia virus DNA genomic PCR amplification and RFLP analysis were described in [38]. Briefly, 20 set of primers were used to amplify the vaccinia virus DNA and PCR mixtures contained ∼ 100 ng of viral DNA and 0.25 μg a primer pair in 50 μL of a reaction. Expand Long Template PCR Kit (Roche Molecular Biologicals, Indianapolis, Indiana, USA) was used with the following conditions: after 2 min at 92 °C for DNA denaturing, reaction mixtures were thermocycled 10 times through successive denaturing (92 °C for 10 s), annealing (55 °C for 30 s), and elongation (68°C for 9 min) steps, and then through 20 cycles of denaturing, annealing, and elongation in which each successive elongation step added 20 s. The amplicons were digested using restriction endonuclease *Bst*UI and the digests were separated by PAGE using commercially available pre-cast 4–20% vertical gels (Invitrogen-Novex, Carlsbad, California, USA) run at 110 V for 140 min in 40 mM Tris-borate (pH 8.0), 1 mM disodium EDTA (TBE) buffer. The gel images were analyzed using the software BioNumerics version 3.5 and the software instructions were used to assess and compare RFLP patterns [46].

## 4. Conclusions

Fowlpox virus has been shown previously to rescue transfected intact and fragmented orthopoxvirus genomes by heterologous packaging [4,24,26]. We have shown that transfected genomic vaccinia virus DNA can be readily rescued and viable vaccinia virus isolated from cells infected with FPV. The background of small plaques predicted to be FPV derived can be further reduced by performing the infection/transfection reaction on the mammalian cell line A549 and subsequent plaque purification on mammalian cell lines. Furthermore, in cells transfected with intact VV DNA and a PCR fragment containing the desired mutation, recombinant VV isolates can be generated at high rates, 21–100%. This method of generating recombinant viruses eliminates the need for selectable markers and traditional plasmid construction and accelerates the process of generating recombinant viruses.

While in general, mutants generated via rescue with a host range restricted helper virus such as FPV are acceptable for *in vitro* studies, we have demonstrated that investigators should be cautious in the use of these viruses for *in vivo* studies. In our study, less than 25% of the resulting recombinant viruses exhibited a phenotype indistinguishable from that of wtVV when examined in mouse pathogenesis studies. Most concerning for this technique but nevertheless interesting in a broader sense is that 33% of the viruses isolated were completely apathogenic in mice and exhibited a corresponding increase in plaquing differential on CV-1 and PK-15 cells. one isolate had almost no alteration in plaquing efficiency in tissue culture but the virus was apathogenic in mice.

## Figures and Tables

**Table 1.** *In vitro* and *in vivo* evaluation of rescued wildtype VV (RwtVV). Isolates were inoculated intratracheally into C57BL/6 mice at 10^6^ or 10^7^ pfu and monitored for severe disease as compared to wtVV infected animals. The viruses were also plaqued on CV-1 and PK-15 cells and the ratio of plaques on CV-1:PK-15 calculated as a measure of viral fitness.

**Virus Isolate**	**Phenotype in Mice**	**CV-1:PK-15 Plaquing Efficiency Ratio**
RwtVV 1	<2 logs attenuated	80
RwtVV 2	<2 logs attenuated	79074
RwtVV 3	wild type	21
RwtVV 4	<2 logs attenuated	275
RwtVV 5	1 log attenuated	20
RwtVV 6	wild type	16
RwtVV 7	2 logs attenuated	1084
RwtVV 8	1 log attenuated	9074
RwtVV 9	1 log attenuated	36
wtVV	wild type	23
